# Calcium Ions as Conjugation-Specific Regulators in *Paramecium caudatum*

**DOI:** 10.3390/microorganisms14020263

**Published:** 2026-01-23

**Authors:** Nobuyuki Haga

**Affiliations:** Department of Biological Sciences, Faculty of Science and Technology, Senshu University of Ishinomaki, Ishinomaki 986-8580, Japan; parame21l@outlook.jp; Tel.: +81-09058355687

**Keywords:** calcium atlas, ciliate conjugation, Ca^2+^/EGTA buffer, microinjection assay, EF-hand fusion kinases, intracellular calcium thresholds, O3 mating substance, *Paramecium caudatum*

## Abstract

The unicellular ciliate *Paramecium caudatum* undergoes a developmental transition from asexual binary fission to sexual reproduction during its mature stage. This transition is triggered by mating interactions between cells of complementary mating types, leading to aggregate formation, mating pairs, and the meiotic division of micronuclei. Although calcium-driven EF-hand kinases have been implicated as mating type proteins, the spatiotemporal dynamics of calcium signaling during conjugation have not been comprehensively characterized. In this study, we established a behavioral assay to isolate committed cells from aggregates immediately after mating onset, and developed an experimental system to monitor intracellular calcium fluctuations specifically expressed in these cells. By combining Ca^2+^/EGTA buffering and microinjection approaches, we manipulated extracellular and intracellular calcium levels and confirmed the continuous requirement of calcium ions for conjugation-specific functions. Two significant findings emerged. First, we identified, for the first time, a calcium atlas covering the entire cell, with ascending centers localized in the anterior, oral apparatus, and posterior regions. The calcium/Indo-1-AM fluorescence peaked at 6 h after mating initiation and declined gradually, but persisted until conjugation was completed at ~48 h. Second, we demonstrated that distinct intracellular calcium thresholds are required for each stage of mating, including maintenance of mating activity, commitment of micronuclei to meiosis, and two-stepwise formation of mating pairs. These thresholds function as regulatory checkpoints that coordinate subcellular localization and stage synchronization. Collectively, our findings highlight calcium ions as pivotal regulators of conjugation in *Paramecium* and propose a novel framework, the *Paramecium* calcium atlas, for understanding the cellular and molecular mechanisms underlying sexual reproduction in ciliates.

## 1. Introduction

Eukaryotes are classified into nine supergroups or supergroup-compatible clades based on genome DNA sequence data and ultrastructural features observed through electron microscopy [1,2,3]. While multicellular eukaryotes are grouped into a limited number of categories within the supergroup, unicellular eukaryotes are spread across all supergroups. To understand the factors that led to the formation of supergroups, it is essential to systematically identify the key characteristics unique to each group and those shared among them. The accumulated knowledge in cell physiology, molecular biology, and ultrastructural morphology has provided essential characteristics for the classification and evolution of unicellular eukaryotes. Alveolata ciliates are one of the most extensively studied groups of unicellular eukaryotes. The most fundamental characteristic shared by eukaryotes is sexual reproduction, and *Paramecium* has the potential to serve as a model organism for studying the origin and evolution of mating patterns and calcium-associated sexuality [4,5].

We focus on genetic information underlying mating-type differentiation and complementary mating-type recognition in sexual reproduction in *Paramecium*. The mating types of *Paramecium caudatum* are determined by a pair of alleles, called odd-mating type gene and even-mating type gene [4]. Recently, the gene responsible for determining the mating type of *Paramecium* has been identified, and its internal structure has been studied [6]. The odd-mating type gene (*Oms3*) encodes a calcium-ion-driven protein kinase fusion polypeptide, with a domain of high homology to protein kinases present in the region of approximately 20 amino acid residues inside the N-terminal methionine and four EF-hand calcium-binding motifs at the C-terminus. Calcium ions act as second messengers to regulate processes in both prokaryotic and eukaryotic kingdoms [7,8]. We have developed an experimental system to investigate the physiological role of calcium ions during conjugation in *Paramecium*.

The mating process begins with random collisions between cells of complementary mating types, leading to adhesion between cilia on the ventral surface of the cell body [4]. Afterward, the mating pairs form close adhesion between two complementary mating-type cells for about 15 h. During this process, the two cells undergo independent micronuclear meiosis, producing two germ nuclei, which are either migratory or stationary pronuclei. The migratory pronucleus is then exchanged between the mating pair and ultimately fuses with the stationary pronucleus of the pair cell to form the genome of the next generation.

The *Paramecium* conjugation process displays several distinct characteristics [9]. The first event, the mating reaction, is a highly specific phenomenon that occurs only between cells of complementary mating types [4,6]. Simultaneously, as mating pairs form, the micronucleus emerges from its storage site within the macronucleus, a process known as early micronuclear migration (EMM) [10], and prepares to enter the premeiotic DNA synthesis phase.

Meiosis of the micronucleus occurs synchronously between the mating cells. The exchange of migratory pronuclei happens alongside the triangular pyramidal structure that forms in the vegetative state of the oral apparatus [11]. Fertilization begins with the fusion of the migratory and stationary pronuclei and proceeds synchronously, taking approximately 48 h to produce a new generation of cells with both a micronucleus and a macronucleus. During this process, four macronuclear anlagen are formed in each mating pair and are distributed among the four cells resulting from two cell divisions. Consequently, the exconjugant begins a new generation with four progeny cells (karyonides). Eight types of karyonides originate from a single mating pair.

A previous study has identified the material basis for the synchronized progression of the conjugation process between mating pairs. We reported that histone H2B messenger RNA is transferred between mating pairs. Using a fusion of the histone H2B gene with the YFP protein gene, we confirmed that histone H2B is imported into the nuclei of all types of mating pairs [12]. In this study, we developed a method to connect the progression of the mating process with changes in calcium ion signals by using a calcium ion fixation technique with a calcium–EGTA (Ca^2+^/EGTA) buffer and measuring changes in intracellular calcium ion concentration using the ratiometric dye Indo-1-AM to monitor calcium ion dynamics throughout conjugation. Additionally, by microinjecting Ca^2+^/EGTA buffer, we estimated the calcium ion threshold needed for the conjugation process.

We gathered valuable information on the dynamic relationship between extracellular fluid and intracellular calcium ions, providing the first comprehensive visualization of temporally and spatially regulated Ca^2+^ signals associated with conjugation in *Paramecium caudatum.* An unexpected discovery in the *Paramecium* calcium atlas was the statistical analysis of calcium fluorescence signal dynamics, which indicated the formation of microdomains between three calcium ion accumulation centers. These results will provide new insights into the role of Ca^2+^ as a crucial regulatory signal in sexual reproduction and the preservation of the calcium signaling machinery overall. We will discuss the features of the calcium signaling system during the evolutionary transition from prokaryotes to eukaryotes.

## 2. Materials and Methods

### 2.1. Paramecium Stocks and Culturing Methods

We used *Paramecium caudatum* stocks Syngen 3, C103 (even mating type, wild-type behavior), 27aG3 (odd mating type, wild-type behavior), and 16B1012 (odd mating type, CNR(*cnrB*) behavior), kindly provided by M. Takahashi. Cultures were prepared following Hiwatashi’s method [4]. Lettuce juice was prepared using K-DS (a buffer solution in which NaH_2_PO_4_ was replaced with KH_2_PO_4_ in Dryl’s solution, pH 7.0 [11]). A 1.25% lettuce juice inoculated with *Klebsiella pneumoniae* was incubated at 25 °C one day before use. To prepare *Paramecium* cultures with over 70% of the cell population exhibiting mating activity, approximately 0.5 mL of *Paramecium* suspension (containing around 800 cells/mL) was added to 2 mL of culture medium on the first day. Then, 4, 8, and 8 mL of culture medium were added over the next three days. The culture medium (stationary growth phase) collected the day after the final addition was used for the experiment [4].

### 2.2. Detection of Intracellular Calcium Ions Using Fluorescent Calcium Indicator, Indo-1 and Indo-1-AM

Indo-1 and Indo-1-AM, used in the experiment, were produced by AAT Bioquest. Inc. (Pleasanton, CA, USA). A stock solution of Indo-1-AM was prepared at 1 mM in 10% DMSO and 100 mM HEPES (pH 7.0). This solution was diluted 100-fold with K-DS (pH 7.0) before use and served as the standard solution in this experiment. To prepare a calibration curve for calcium ion concentration using Indo-1 in vitro, Indo-1 was used instead of Indo-1-AM as the stock solution.

The fluorescence intensity of calcium ion-bound and unbound Indo-1-AM was measured using two dichroic mirror filters: calcium-bound: DM-400 nm, EX-360 nm, and BA-420 nm; unbound: DM-380 nm, EX-360 nm, and BA-460 nm. Under these measurement conditions, the bound and unbound filters were manually switched to detect two fluorescence types in the same cell continuously. The cells were photographed using a Nikon FX-35DX camera with exposure control from the Nikon UFX-II [12]. The exposure time was fixed at 2 s for all images. To ensure the cells were alive and motionless, the extracellular fluid volume was adjusted with a micropipette without a cover glass.

### 2.3. Quantifying Relative Fluorescence Intensity Through Computer Image Analysis

The images captured with the fluorescence microscope were transferred to a computer, and the fluorescence intensity was measured in Photoshop. Three regions of the cell body—the anterior, the oral apparatus region, and the posterior—were scanned consecutively with a cadelart (0.4 × 0.4 µm), and the maximum, average, and standard deviation of fluorescence intensity were obtained from the pixel histogram for each region.

### 2.4. Calcium Ion Concentration Clamp Method

Experiments in which extracellular and intracellular calcium levels were artificially adjusted used Ca^2+^/EGTA buffers. The clamped-free calcium ion concentration was calculated based on the equilibrium constant at 23 °C and pH 7.0 [13].

### 2.5. Microinjection of Ca^2+^/EGTA Buffers

Microinjection was performed using the Koizumi method [14], modified by Haga et al. [15]. To adjust the intracellular free calcium ion concentration, 40 pL of Ca^2+^/EGTA buffer solution at various concentrations was injected, equivalent to approximately 10% of the cell volume.

### 2.6. Statistical Analysis

The data were tested for significance using various statistical methods as needed. One-way ANOVA followed by Tukey’s Honest Significant Difference (HSD) test for multiple comparisons. Welch’s *t*-test computed from summary statistics (mean, SD, *n*) and adjusted by the Holm method. The analysis was performed on group means across different regions (Anterior, Oral apparatus, central, and Posterior) and time points (0, 1, 5, 30, and 60 min). Two-sided *p*-values were Holm-adjusted within each time slice to control the family-wise error rate. Effect sizes were reported as Hedges’ g with small-sample correction. Because raw observations were unavailable, *p*-values and effect sizes are approximations derived from summary-level inputs. A *p*-value <0.05 was considered statistically significant in all statistical analyses.

## 3. Results

### 3.1. The Method for the Isolation of Mating-Competent Cells

**Figure 1:**
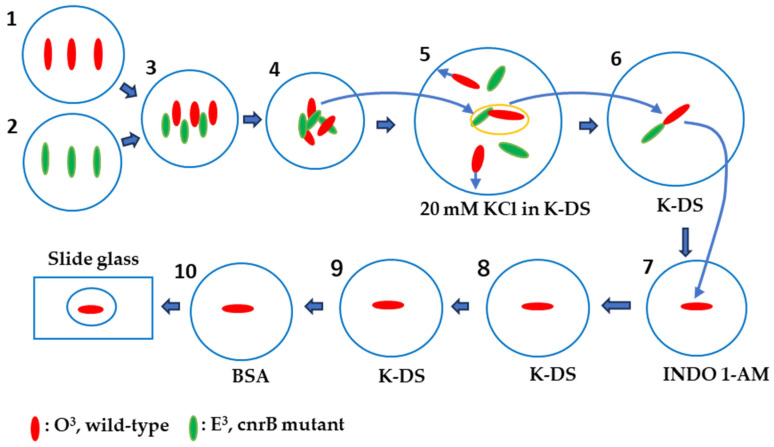
Isolation of single cells committed to the mating reaction and the Indo-1-AM administration method. The red oval indicates an odd-mating type of Syngen 3 with wild-type behavioral traits, while the green oval indicates an even-mating type of Syngen 3 with CNR (caudatum-non-reversal mutant) behavioral traits. The numbers in the figuer indicate the experimental procedure. The detail of each step are explained in the results text.

When cells of complementary mating types expressing mating activity are mixed, cell aggregates form. Since the mating reaction of *Paramecium* was reported in 1968, it has been thought that each cell in the aggregate enters the conjugation process synchronously. However, in this experiment, we found that the calcium fluorescence intensity at the cell surface was not synchronized among cells within the aggregate. Therefore, we developed a method to isolate the most strongly sexually bonded cell pairs from cell aggregates, thereby obtaining statistically precise data. We used a behavioral mutant, *cnrB*, which lacks the function of the voltage-dependent calcium channel in the ciliary membrane. When this mutant is transferred to K-DS containing a high concentration of potassium ions (20 mM KCl), the ciliary beating does not reverse, and the cells remain stationary. In contrast, wild-type cells are induced to reverse their ciliary beating and start swimming backward quickly (for approximately 1 min). Figure 1 shows the method for isolating the tightly bounding mating pairs among the mating clumps. 1. Mating type: O type (Red), swimming behavior: wild type. 2. Mating type: E type (Green), swimming behavior: CNR mutant. 3. The two strains were mixed. 4. Cell aggregates were formed due to ciliary adhesion between complementary mating cells. 5. One mating clump was sucked with a micropipette and transferred to the backward swimming induction solution (20 mM KCl in K-DS). Cells that adhered to the heads remained in the central area of the solution. Arrows indicate the direction of backward swimming in wild-type cells, while the mutant slowly turns in place. 6. Using a micropipette, the cell pairs were sucked and transferred to the K-DS. 7. The wild-type cells that no longer attached to the head were sucked up with a micropipette, transferred to the Indo-1-AM solution, and incubated for 20 min. 8. The cells were transferred to the K-DS solution and allowed to stand for 30 s. 9. The cells were transferred to a fresh K-DS and 10. Finally, the cells were transferred to the BSA solution and placed immediately on glass slides. The BSA solution was sucked until just before the cells stopped swimming. The cells were observed using a fluorescence microscope, and photographs were taken for documentation.

### 3.2. Calcium Indo-1-AM Measurement and Quantitative Assay

**Figure 2:**
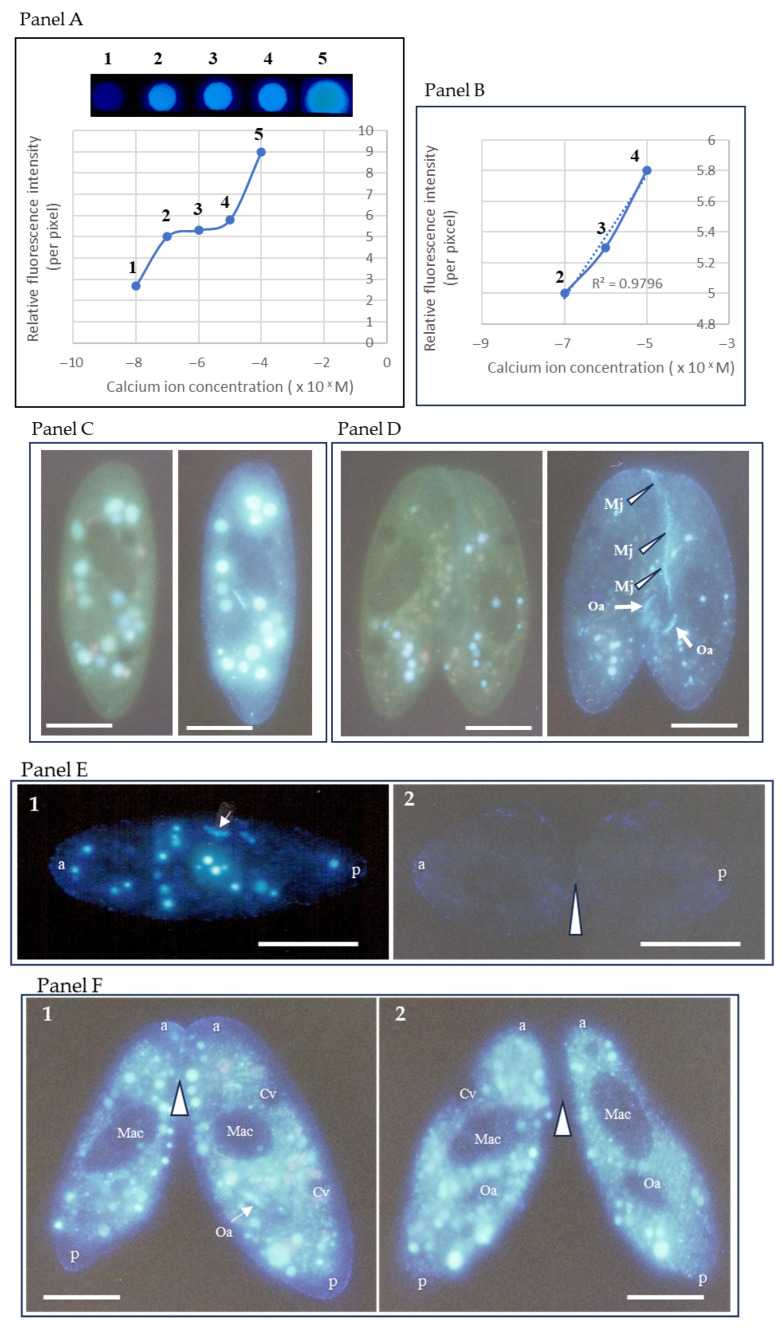
Quantitative analysis of intracellular calcium ion levels using Indo-1 and Indo-1-AM. (**A**) Fluorescence signal intensity of Indo-1 and calcium ion concentration. The fluorescence signals numbered 1–5 at the top correspond to the relative fluorescence intensities shown in the graph below. The horizontal axis represents the free calcium concentration (10^−^^x^ M) of the Ca^2+^/EGTA buffer, while the vertical axis indicates the relative fluorescence intensity. (**B**) Enlarged graph showing the concentration range from 2 to 4 in (**A**). (**C**) Calcium fluorescence signal of a cell one minute after the start of the mating reaction. The left panel shows the intracellular fluorescence signal of unbound Indo-1-AM, while the right panel shows the intracellular fluorescence signal of calcium ion-bound Indo-1-AM. Images of the same living cell were taken consecutively with an exposure time of 2 s. The particulate fluorescent vesicles in the cytoplasm are calcium fluorescence within food vacuoles. (**D**) A mating pair 60 min after the onset of the mating reaction. The white arrow Oa indicates the oral apparatus, and the white arrowhead Mj indicates the mating junction. (**E**) Indo-1-AM uptake in cells during the mating reaction, and a dividing cell incubated in Indo-1-AM solution. In (**E1**), a cell in the mating reaction displayed the formation of approximately 20 fluorescent food vacuoles within 20 min of incubation. The white arrow indicates the oral apparatus. “a” is anterior, and “p” is posterior. In contrast, the cell during the dividing phase, shown in (**E2**), did not exhibit any food vacuoles, and the fluorescence observed at the cell surface was faint. Consequently, an image of the dividing cell was captured with an exposure time of 4 s. A white arrowhead indicates the fission furrow. “a” indicates anterior, “p” posterior. The bar indicates 50 µm. (**F**) Comparison of fluorescence intensity near the cell surface when Indo-1-AM is administered extracellularly (**F1**) and microinjected into cells (**F2**). “a” and “p” are anterior and posterior, respectively. Mac indicates a macronucleus, Cv is a contractile vacuole, and Oa is an oral apparatus. A white arrowhead shows the mating junction between the mating pair. The bar indicates 50 µm.

Panels A and B in Figure 2 show the results of an assay to quantitatively evaluate calcium ion dynamics in *Paramecium* cells using Indo-1 and Indo-1-AM. Once inside the cell, it is converted to the free acid form by endogenous esterases, which emits a fluorescent signal upon calcium ion binding. All experiments were performed under a strictly identical loading protocol across conditions, minimizing systematic differences in dye uptake, compartmentalization, or potential leakage. The calcium ion-bound form emits white fluorescence, and its fluorescence intensity increases proportionally with calcium ion concentrations (Figure 2A). The fluorescence intensity resulting from Indo-1 binding to calcium ions showed a strong positive correlation with calcium ion concentrations ranging from 200 nM to 20 µM (Figure 2B). The conjugation properties, including mating activity, migration of micronuclei from the macronuclear pocket (EMM), and mating pair formation rate, were assessed within this concentration range (Figure 2B).

Indo-1-AM is taken up by the cell from the extracellular solution and spreads evenly throughout the cell (Figure 2C,D). Two types of fluorescent signals were observed in the cytoplasm: one from the indicator, Indo-1-AM, inside food vacuoles via the oral apparatus, and the other from the indicator absorbed through the cell membrane into the alveolar sac (Figure 2C). In the conjugated pair, 60 min after the start of conjugation, a strong fluorescent signal was detected at the mating junction (three white arrowheads in Figure 2D, the photograph of the right side). Figure 2E shows the comparison of calcium ion signals during cell division in the vegetative growth phase (Figure 2E2) and immediately after the start of the mating reaction (1 min) (Figure 2E1). To clarify the pathway by which Indo-1-AM is taken up into cells, we conducted experiments using extracellular administration and intracellular microinjection. When Indo-1-AM was added externally, calcium fluorescence signals were clearly visible at the mating junction of the mating pairs and on the cell surface (Figure 2F1). No signal was detected in the macronucleus or contractile vacuoles. Many bright vesicles appeared in the cytoplasm. In cells injected internally via microinjection, no fluorescence was observed on the cell surface or at the mating junction (Figure 2F2). A common feature of both extracellular and microinjection administration is that calcium signals in the macronucleus and contractile vacuoles were below the detection limit. However, the fluorescence intensity in microinjected cells was below the detection limit in two areas: the mating junction and the oral apparatus. These findings suggest that the supply of Indo-1-AM to the mating junction and oral apparatus relies on an extracellular pathway.

### 3.3. Characterization of the Paramecium Calcium Atlas

**Figure 3:**
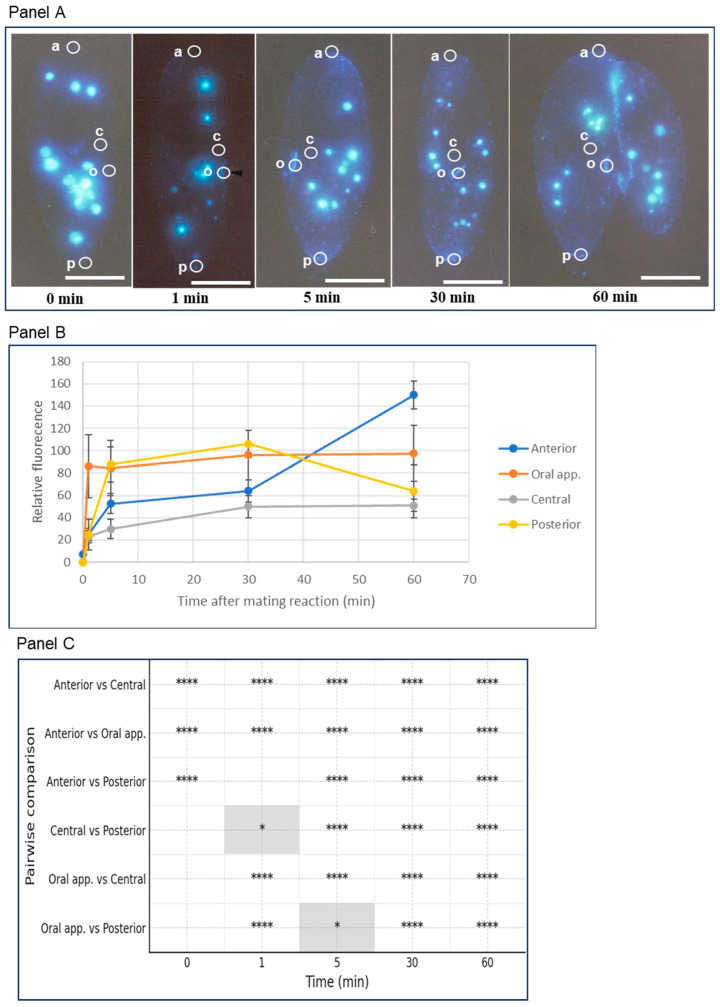
Photographs showing intracellular calcium ion fluorescence from the start of the mating reaction to the formation of the holdfast union. (**A**) shows calcium–Indo-1-AM fluorescence during the early stages of conjugation. 0 min marks the time before the mating reaction begins, while 1–60 min indicates the time after it starts. The white circles highlight the areas where fluorescence intensity was measured. “a” represents the anterior region, “c” the central region, “o” the oral apparatus region, and “p” the posterior region. The light blue globules scattered in the cytoplasm are the fluorescence of Indo-1-AM calcium that has been taken up into the food vacuole. The fluorescence of food vacuoles is not included in the graphs in (**B**). The white bar indicates 50 µm. Panel B shows the change in fluorescence intensity in four regions of the cell (anterior, oral apparatus, central, and posterior). The vertical axis indicates relative fluorescence intensity, and the horizontal axis shows time from the start of the mating reaction. Bars represent the standard deviation. The significance test results between regions at each time point are displayed in (**C**). *p*-values were calculated using Welch’s *t*-test from summary statistics (mean, standard deviation, *n*) and adjusted with Holm’s method at each time point. Blank spaces indicate areas with no significant differences. * *p* < 0.05, **** *p* < 0.0001.

The calcium atlas describes changes in calcium signals from the start of mating to the end of conjugation, which lasts about 48 h. The main feature of this graph is that the fluorescence intensity, which indicates changes in calcium ion levels, shows different patterns in each of the four regions during the 1–60 min after the onset (Figure 3A–C). One minute after the mating reaction began, the fluorescence signal from the oral apparatus region increased sharply and stayed at the same peak level for 60 min. Five minutes later, the fluorescence signal from the posterior region increased, remained at the same peak level as the oral apparatus for 30 min, and then decreased to a low central level after 60 min. The fluorescence signal from the anterior region remained at a low central level for 30 min, then increased sharply, reaching about 5 times the minimum central level after 60 min. The relationship between the cell’s fine structure and the physiological effects of threshold concentrations will be discussed.

### 3.4. Comparison Between the Characterization of the Calcium Atlas at 1 and 6 h Conjugating Pairs

**Figure 4:**
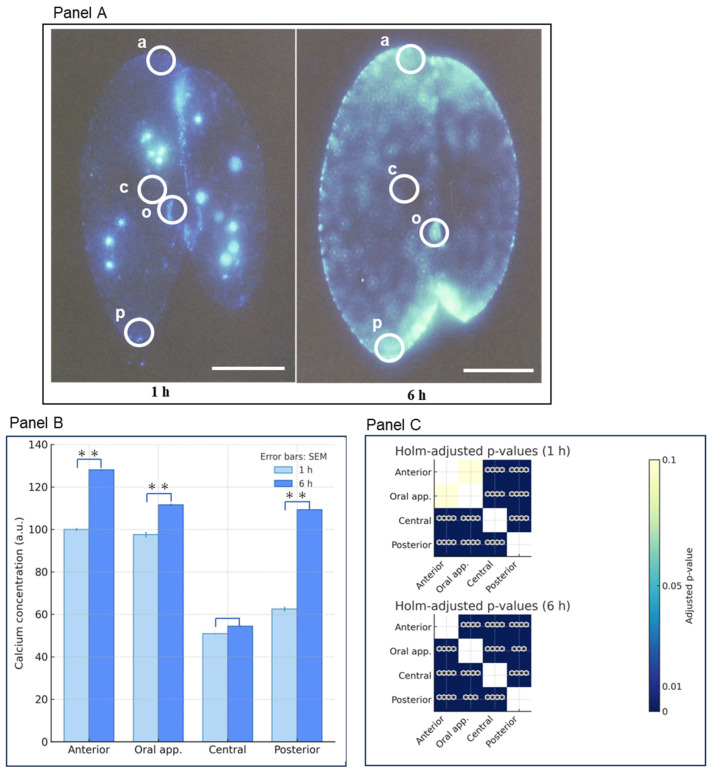
Comparison of intracellular calcium fluorescence intensity in holdfast and paroral adhesions after adhesion onset. (**A**) Fluorescence images taken 1 h (left) and 6 h (right) after adhesion onset. White circles indicate the areas where fluorescence intensity was measured. “a” represents the anterior region, “c” the central region, “o” the oral apparatus region, and “p” the posterior region. The white bar indicates 50 µm. (**B**) shows graphs comparing fluorescence intensity at four sites. The vertical axis shows the mean and standard error of the relative fluorescence intensity. The horizontal axis shows the four sites. ** indicates *p* < 0.01. (**C**) displays a heat map of Holm-adjusted *p*-values between regions for 1 h mating pairs (top panel) and 6 h mating pairs (bottom panel), respectively. Both panels show four regions on the vertical and horizontal axes. Significance tests for fluorescence intensity between regions are determined as Holm-adjusted *p*-values. Light yellow and white columns indicate no significant differences. *** indicates *p* < 0.001, and **** indicates *p* < 0.0001. White boxes indicate *p* ≥ 0.1.

Comparing the calcium concentration increase from 1 h to 6 h, the central region showed a suppressed rise, while the other three areas exhibited significant increases (Figure 4A–C). The rate of increase was highest in the posterior region. The final concentrations were anterior > oral apparatus > posterior, but the rate of increase was greater in the posterior region compared to the anterior and oral apparatus regions. The heat map in panel C displays the significance test results for fluorescence intensity between the regions at 1 and 6 h, respectively. After 1 h, no significant difference was observed between the anterior and oral apparatus. Significant differences were observed between all other regions. This suggests the existence of calcium storage microdomains within the cells.

### 3.5. Determination of the Calcium Ion Threshold for the Conjugation Process Using the Calcium Ion Fixation Method with Ca^2+^/EGTA Buffers

**Figure 5:**
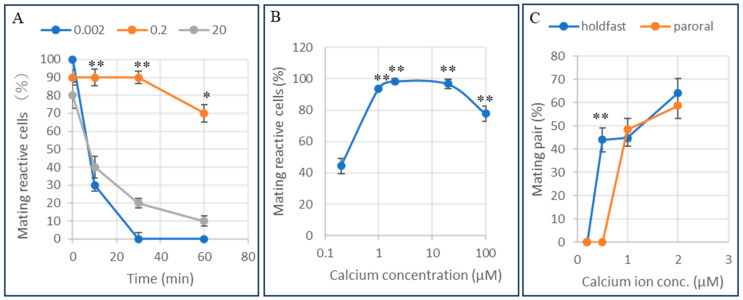
(**A**) Effect of Ca^2+^/EGTA microinjection on maintaining mating activity. Cells exhibiting mating activity were injected with various concentrations of Ca^2+^/EGTA (0.002, 0.2, and 20 µM). The vertical axis is the percentage of cells showing mating activity. The horizontal axis is the time after microinjection. Data are presented as mean ± SD (*n* = 10 per group). Statistical comparisons were conducted using Tukey’s multiple-comparison test, which revealed significant differences between 0.2 µM and all higher concentrations. The mark * indicates *p* < 0.05, and ** indicates *p* < 0.01. (**B**) Effect of extracellular Ca^2+^ concentration on the maintenance of mating reactivity. (**C**) Effect of external calcium ion concentration on mating pair formation. The first stage is holdfast union, and the second stage is paroral union. The graph shows the percentage of each type of mating pair 60 min after the external concentration was set. The vertical axis represents the percentage of mating pairs, and the horizontal axis represents the external calcium ion concentration.

The threshold concentration of intracellular calcium ions that influences the maintenance of mating reactivity was examined. At the start of the experiment, mating-reactive cells were isolated from the culture medium using the method shown in Figure 1. Then, a solution with the specified free calcium concentration (Ca^2+^/EGTA buffer) was injected at a volume equal to 10% of the cell’s volume, and mating reactivity was monitored over time. As shown in Figure 5A, a specific concentration of calcium ions is required intracellularly to maintain mating reactivity. 10 min after injection, cells maintained mating reactivity over the range of 0.002 to 20 µM. The calcium ion concentration tolerance required to sustain mating reactivity is effective over a range of tens of micromolar.

Next, the effect of extracellular calcium ion concentration on mating reactivity was examined. To use a population with a 100% expression rate for the assay, reactive cells were selected using the method shown in Figure 1. They were incubated in Ca^2+^/EGTA buffers at various concentrations. After 60 min, the cells were harvested, and the percentage of cells that retained mating reactivity was measured using a tester strain of cells of the complementary mating type. As shown in Figure 5B, the calcium ion concentration required to maintain mating reactivity was 1–100 µM. After the mating reaction, cell aggregates form, and mating pairs appear 30–60 min later.

The extracellular calcium ion concentration necessary for mating pair formation was examined (Figure 5C). The calcium ion concentrations required for the formation of holdfast and paroral unions were 0.5 µM and 1.0 µM, respectively, indicating a positive correlation between the calcium ion concentration in the extracellular fluid and the stepwise progression of mating pair formation.

Cells were incubated in medium containing 0.2–100 µM Ca^2+^. The vertical axis is the percentage of mating-reactive cells. The horizontal axis is the extracellular concentration of calcium ions. Data are presented as means ± SD (*n* = 12 per group) using Tukey’s multiple comparison test. ** indicate *p* < 0.01. Figure 5C, Effect of extracellular free calcium concentration on the formation of holdfast union and paroral union. The percentage of holdfast union (blue) and paroral union (red) was measured at various concentrations of Ca^2+^ (0.2–2 µM) at 60 min after the treatments. The vertical axis is the appearance rate of the mating pair, and the horizontal axis is the extracellular free calcium ion concentration. The holdfast union was induced at 0.5 µM, while the paroral union required 1.0 µM for formation. Data are expressed as mean ± SD, and statistical comparisons were performed using Welch’s *t*-test. The mark ** indicates *p* < 0.01.

### 3.6. Summary of the Paramecium Calcium Atlas

**Figure 6:**
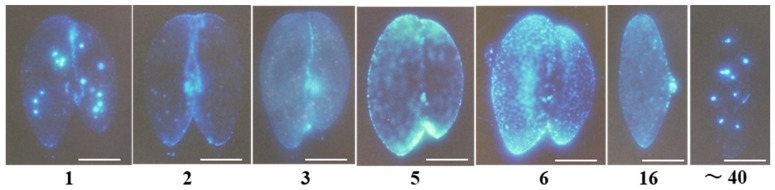
Changes in calcium signals over time in mating pairs. The numbers below each photograph indicate the time (hours) since mating began. Photograph 16 shows a cell immediately after separation of the mating pair. Photograph 40 is called a karyonide, the beginning of an offspring. The white line indicates 50 μm. All of the pale particles within the cells are derived from calcium ions bound to Indo-1-AM. However, photos 1 and 40 also contain color from food vacuoles. The pale particles in photos 2–16 are derived from intracellular calcium storage organelles.

The morphological features of calcium signal emitters vary clearly across the four regions: anterior, oral apparatus, posterior, and mating junction (Figure 6). The fluorescent emitters in the oral apparatus region are short, curved, string-like, and notably independent from the surrounding areas (photographs 1 and 40). The fluorescent signals at the front and tail of the cell form gentle gradients from the apex of the triangular pyramid toward the periphery at each end (photographs 2, 5, and 6). The fluorescent signal emitters in the mating junction of the conjugates are granular and arranged in a linear pattern (photographs 1, 2, and 3). In cells, 5 to 16 h after conjugation begins, fluorescent bodies appear to be arranged in layers across the entire cell surface. The triangular area protruding to the right in photograph 16 is the mouth region, which is a depression structure in the cytoplasm during the growth phase. At the later stage of conjugation, it turns outward to the outside of the cell. This is the first time strong fluorescence has been observed in this region. We speculate that calcium ions might play a role in positioning the migrating nucleus and transporting it to the partner cell.

Table 1 summarizes the sequence of events over time in the cilia on the cell surface, the cytoplasm, the cell membrane, and the micronucleus and macronucleus, with the minimum calcium ion concentration required for each stage of conjugation serving as the threshold. Micronuclei progress from the premeiotic DNA synthesis stage to the meiotic stage, exhibiting morphological changes. The primary morphological changes include swelling, crescent, elongation, and globular stages.

**Figure 7:**
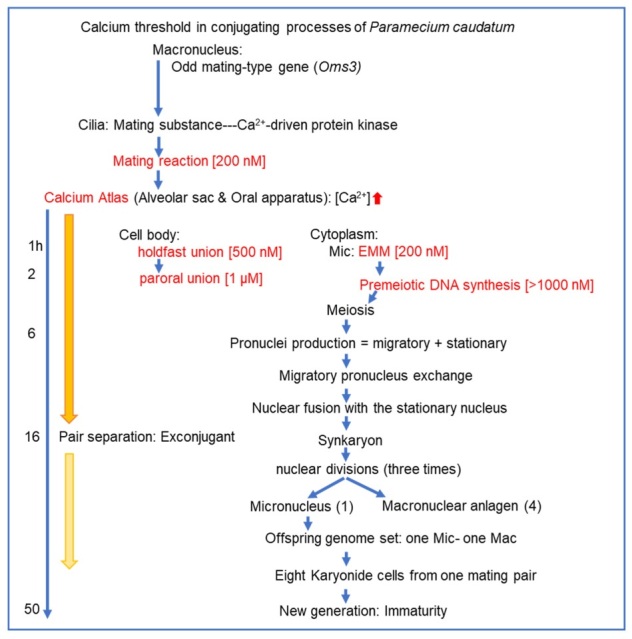
Summary of the calcium threshold estimated during the conjugation process. The phenomena written in red are findings confirmed in this study. The concentrations indicated in [] represent the minimum external calcium ion concentration required for the reaction to occur. Below this concentration, the conjugation process stops. The downward-pointing arrow in the calcium atlas qualitatively indicates a decrease in calcium concentration approximately 16 h after the mating reaction begins. The numbers 1 and 4 in parentheses indicate the number of micronuclei and macronuclear anlagen, respectively. The blue arrow indicates the direction of time. The red arrow indicates an increase in intracellular calcium concentration. The yellow arrows qualitatively indicate the change in intracellular calcium concentration.

To clarify the calcium ion threshold during the conjugation process over time, the data points reported in this study are shown in Figure 7. The causal relationship between the increase in calcium ion concentration throughout the conjugation process and the chemical reactions occurring during it remains only partially clear. We hope that this figure will provide valuable clues for developing future analytical strategies.

## 4. Discussion

This report demonstrates that sexual reproduction in *Paramecium* proceeds through a signaling loop, with calcium ion concentration thresholds controlling the activation of key steps in the early stages of conjugation. This is the first time that calcium ions are more than just second messengers; they are involved in a series of mating-specific events through the conjugation processes.

The calcium atlas in *Paramecium caudatum* was identified using the ratiometric calcium indicators Indo-1 and Indo-1-AM, characterized by a timeline and spatial locations. The timeline was divided into two phases: an early phase (0–60 min) and a middle phase (1–6 h). Statistical analysis of calcium ion concentration changes was conducted for each period. The calcium ion concentration range in the calcium atlas is in the millimolar range, whereas the effective threshold concentrations of extracellular and intracellular calcium are in the nanomolar range. The relationship between calcium ion reserves and optimal functional concentrations observed in our study aligns with the characteristics of ionized calcium reserves reported in many organisms [16].

Our experimental system revealed two unique features: first, the rates of increase, decrease, and steady-state changes in calcium ion concentration fluctuate independently among compartmentalized regions within the cell, and second, the altered state persists for an extended period, ranging from several hours to several tens of hours. The maximum calcium atlas concentration was in the high range, exceeding 200 µM. However, functional inhibition caused by microinjection and by external solutions containing Ca^2+^/EGTA buffers revealed that the effective concentrations ranged from 0.1 to 20 µM. Initial steps in the conjugation process require a threshold increase from about 0.1 to 0.5 µM, indicating a calcium-ion threshold-controlled switch that triggers conjugation.

The calcium indicator that binds calcium ions will alter local calcium concentration [16]. To minimize this physiological effect on the conjugation process, the incubation time in the indicator solution was kept as short as possible to detect the fluorescent signal. To reduce nonspecific binding to the cell surface, paramecia were washed with physiological saline before use. The aperture value and exposure time for photographing with a fluorescent microscope were kept constant for all photographs. This method showed a strong link between the fluorescent intensity in solution and the calcium ion concentration.

**Figure 8:**
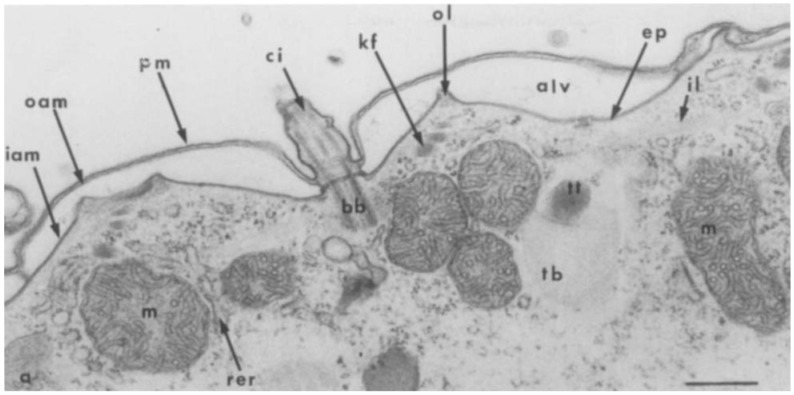
Ultrastructure of *Paramecium* cortex and sites of antimonate deposits. The surface of *Paramecium*, as observed in conventional EM sections (a), consists of a continuous plasma membrane (pro) subtended by a single layer of large, tightly apposed membrane vesicles, the alveoli (alv), which are interrupted only at the points of implantation of cilia (ci) through their basal bodies (bb) and trichocysts (n, trichocyst tip; tb, trichocyst body). The alveoli are surrounded by an outer alveolar membrane (oam) in topological continuity with an inner alveolar membrane (iam). They are subtended continuously by a membrane skeleton, the epiplasm (ep). The epiplasm itself is interrupted at the ridges of the alveoli, giving way to filaments known as the outer lattice (ol). Deeper in the cytoplasm, the filaments of the infraciliary lattice (il) are found, running across the rough endoplasmic reticulum (rer) and the mitochondria (m). When cells are preincubated in anfimonate (b), dense deposits are observed within the alveoli and trichocyst tips (arrows). Both cross sections (central alveoli) and more tangential ones (rightmost alveolus) show the deposits in alveoli preferentially located over the inner alveolar membrane. A few grains are also visible in mitochondria. Bar, 0.5/~M. (Figure 8. Ultrastructure of *Paramecium* cortex and sites of antimonate deposits. Reproduced from Stelly et al., J. Cell Biol. 1991, 113, 103–112, under the RUP noncommercial scholarly reuse policy [22]).

Two potential calcium storage structures in the paramecia are the alveolar sac system and the trichocyst. The compartmentalization of calcium ions throughout the cell may result from the presence of various specialized functional molecules. These include ion pumps, ion channels, and ion-binding peptides that store and transport calcium ions [17,18,19,20,21,22]. We focused on the role of the alveoli as organelles where these molecules could be organized to function cooperatively. Calcium ions are expected to function in the conjugation process as follows: 1. induce the expression of mating reactivity to be localized to the anterior-ventral region, 2. promote binding with complementary mating-type cells, 3. induce the degeneration of cilia involved in binding, 4. induce loose primary adhesion between cell membranes, 5. sequentially activate strong adhesion molecules (proteinaceous), and 6. promote long-lasting, stable cell–cell binding and partial cell membrane fusion, creating bridges for pronuclear transport. This indicates that the calcium ion concentration is not solely determined by diffusion but is also actively regulated by changes in calcium uptake, storage, and release. The alveolar sac is a flattened structure located between the cell membrane and the microtubule network, a unique feature of this supergroup that gives the Alveolata its name (Figure 8) [23].

**Figure 9:**
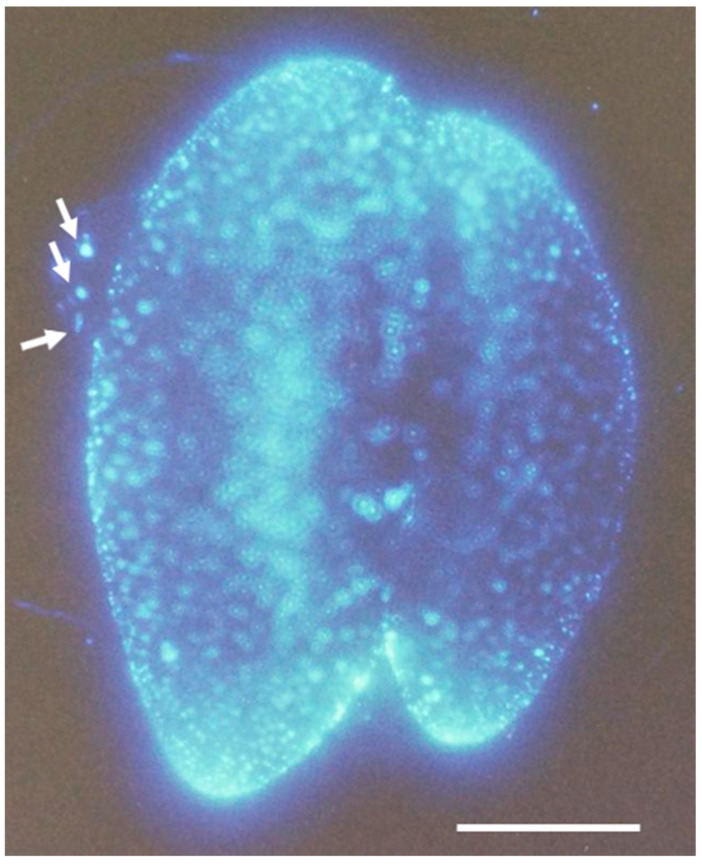
A photograph of the regional distribution of calcium fluorescence signals in conjugating *Paramecium*, measured 6 h after the onset. Three white arrows point to calcium fluorescence particles within the extracellularly exposed membrane-sac protrusion. The white bar shows 50 µm.

No findings have been reported regarding the dynamics of calcium ions within the alveolar sac during the conjugation process. Figure 9 shows one of the images we observed. When cells approach a dry state on a glass slide, membrane-sac protrusions often form on the cell surface. Particulate fluorescent materials were observed within these membrane-sac protrusions. Whether this membrane-bound structure originates from the cell membrane or the alveolar sac remains to be determined.

Many studies have reported on the role of calcium ions in physiological functions, including their impact on alveolar sacs [23,24,25,26,27,28,29,30]. Assuming that the alveoli are functionally compartmentalized, molecular groups that enable functional differentiation may include Ca^2+^ pumps/exchangers, release channels, luminal buffers, and the cortical lipid skeleton. Regarding local calcium ion increases, the targets to be investigated include: 1. Ca^2+^ transport and gating, 2. regulation of intraluminal Ca^2+^ buffering, 3. diffusion barriers formed by the intracellular membrane system and cell surface scaffolds, 4. compartmentalization of signaling platforms by lipid microdomains, 5. regulation of store drive by pH and proton pumps, and 6. control of calcium ion flux between alveolar subcompartments via structural “valves”.

According to recent comprehensive studies, the process of regulating physiological functions through calcium-driven kinases involves a three-step molecular mechanism: first, calcium ions bind to the EF-hand motif, then the kinase shifts from an inactive to an active state, and finally, phosphorylation of the kinase’s substrate protein occurs, causing it to undergo a conformational change and leading to a change in biological function [31,32]. The structures of the EF-hand motifs containing 11–14 amino acid residues in the Ca^2^^+^-binding loop are analyzed within the framework of the recently proposed two-step Ca^2+^-binding mechanism.

The hypothetical Last Common Eukaryotic Ancestor (LECA) is defined as the organism that is the ancestor of all modern eukaryotes, including animals, plants, fungi, and single-celled organisms. Comparative genomics reveals that LECA possessed at least a dozen Ca^2+^-related components, including ER storage, EF-hand groups, and the calcineurin complex [7]. Some EF-hand groups likely originated from eubacterial symbionts, while calcineurin subunits are thought to have originated from *Asgard archaea*, a member of Archaea. Calcium-dependent protein kinases (CDPKs) are found mainly in plants [33], algae, and alveolates [34], but are largely absent in animals, fungi, and bacteria. The presence of CDPKs in the LECA is debated, with questions surrounding their origin and possible convergent evolution.

The fusion gene of calmodulin and kinase genes can serve as an indicator of ongoing evolution from prokaryotes to eukaryotes. Understanding this gene fusion and the developmental progression of the intracellular membrane system, calcium stores, and inter-organelle connections will help form an intracellular calcium regulatory circuit. This eventually contributed to the formation of the LECA.

In summary, 1. Calcium-ion signaling control during sexual reproduction in unicellular eukaryotes is a key innovation in eukaryotic cell evolution from prokaryotes. 2. While there is evolutionary continuity with prokaryotes in calcium-binding proteins and membrane transport systems, the calcium-driven-type internal control system in sexual reproduction is considered a unique evolution to eukaryotes. 3. Though continuity exists at the molecular component level, evolution as a functional network is seen as discontinuous.

## 5. Conclusions

We successfully visualized intracellular calcium dynamics during conjugation in *Paramecium* using the fluorescent indicator Indo-1-AM. Our observations revealed stage-specific spatiotemporal patterns of Ca^2+^ distribution, including early punctate hotspots, cortical concentration at cell–cell interfaces, redistribution along the anterior–posterior axis, and a central decrease at later stages. These findings demonstrate that calcium ions are indispensable for orchestrating multiple steps in the conjugation process, including partner recognition, mating pair formation, germ nuclear production, and fertilization.

The present work establishes the first “calcium atlas” of ciliate conjugation. By providing direct experimental evidence of calcium’s multifaceted roles, this study opens new avenues for mechanistic investigations into the downstream effectors and molecular pathways of calcium signaling in unicellular organisms. Additionally, our results highlight the significance of *Paramecium* as a model for exploring the evolutionary origins of calcium-dependent regulation in sexual reproduction.

## Figures and Tables

**Table 1:** Calcium ion threshold concentration for each stage of conjugation.

	Cell Surface		Nucleus	
Time (h)	Cilia/Cytoplasm	Somatic membrane	Mic/Mac	Ca^2+^-threshold (nM)
0	mating-reactive			200
0.5			Mic: EMM	200
	Cilia: degeneration			nd
1		holdfast ad-site		500
2		paroral ad-site		1000
3			Mic: pmDNA s	>1000
4			Mic: elongation	nd
5	Oa: degeneration			nd
6	Loss of fv formation			nd

Mic, micronucleus; Mac, macronucleus; Oa, oral apparatus; pmDNA s, premeiotic DNA synthesis, ad-site, adhesion site; fv, food vacuole; nd, not detected.

## Data Availability

The original contributions presented in this study are included in the article. Further inquiries can be directed to the corresponding author.
